# MutLγ promotes repeat expansion in a Fragile X mouse model while EXO1 is protective

**DOI:** 10.1371/journal.pgen.1007719

**Published:** 2018-10-12

**Authors:** Xiaonan Zhao, Yongwei Zhang, Kenneth Wilkins, Winfried Edelmann, Karen Usdin

**Affiliations:** 1 Section on Gene Structure and Disease, Laboratory of Cell and Molecular Biology, National Institute of Diabetes, Digestive and Kidney Diseases,National Institutes of Health, Bethesda, MD, United States of America; 2 Department of Cell Biology, Albert Einstein College of Medicine, Bronx, NY, United States of America; 3 Office of Clinical Research Support, Office of the Director, National Institute of Diabetes, Digestive and Kidney Diseases, National Institutes of Health, Bethesda, MD, United States of America; University of Washington School of Medicine, UNITED STATES

## Abstract

The Fragile X-related disorders (FXDs) are Repeat Expansion Diseases resulting from an expansion of a CGG-repeat tract at the 5’ end of the *FMR1* gene. The mechanism responsible for this unusual mutation is not fully understood. We have previously shown that mismatch repair (MMR) complexes, MSH2/MSH3 (MutSβ) and MSH2/MSH6 (MutSα), together with Polβ, a DNA polymerase important for base excision repair (BER), are important for expansions in a mouse model of these disorders. Here we show that MLH1/MLH3 (MutLγ), a protein complex that can act downstream of MutSβ in MMR, is also required for all germ line and somatic expansions. However, exonuclease I (*EXO1*), which acts downstream of MutL proteins in MMR, is not required. In fact, a null mutation in *Exo1* results in more extensive germ line and somatic expansions than is seen in *Exo1*^*+/+*^ animals. Furthermore, mice homozygous for a point mutation (D173A) in *Exo1* that eliminates its nuclease activity but retains its native conformation, shows a level of expansion that is intermediate between *Exo1*^*+/+*^ *and Exo1*^*-/-*^ animals. Thus, our data suggests that expansion of the FX repeat in this mouse model occurs via a MutLγ-dependent, EXO1-independent pathway, with EXO1 protecting against expansion both in a nuclease-dependent and a nuclease-independent manner. Our data thus have implications for the expansion mechanism and add to our understanding of the genetic factors that may be modifiers of expansion risk in humans.

## Author summary

The Fragile X-related disorders arise from expansion of a tandem repeat or microsatellite consisting of CGG-repeat units. The expansion mutation is not well understood, but our previous data suggests that MutSα and MutSβ, mismatch repair (MMR) proteins that normally protect the genome against microsatellite instability, are actually responsible for these mutations in a knockin mouse model of these disorders. In this manuscript we describe the role in expansion of two proteins that act downstream of the MutS proteins in MMR, MutLγ and EXO1. Our data suggests that expansion occurs via a MutLγ-dependent, EXO1-independent pathway, with EXO1 playing both a nuclease-dependent and a nuclease-independent role in preventing expansions.

## Introduction

The 5’ end of the human *FMR1* gene (MIM* 309550) contains an unstable CGG/CCG-repeat tract. This instability shows a strong expansion bias, with alleles having 55–200 repeats, known as Premutation (PM) alleles, being as much as 10 times more likely to expand than contract [1]. The likelihood of expansion increases with increasing repeat number [1]. PM alleles confer risk of a neurodegenerative condition known as Fragile X associated tremor/ataxia syndrome (FXTAS; MIM# 300623) and a form of female infertility known as Fragile X-associated primary ovarian insufficiency (FXPOI; MIM# 300624) [2]. Expansion is seen in somatic cells and in the germline, where it can produce alleles with >200 repeats. Such full mutation (FM) alleles result in Fragile X syndrome (FXS; MIM# 300624), a disorder whose major symptoms include intellectual disability (ID) and autistic behaviors [2]. Collectively these three clinical consequences of CGG/CCG-repeat expansion in the *FMR1* gene constitute the Fragile X-spectrum disorders or Fragile X-related disorders (FXDs). These disorders belong to a larger group of genetic disorders known as the Repeat Expansion Diseases, that all result from an expansion of a tandem repeat in a disease-specific gene. However, whether these diseases share a common expansion mechanism is unclear.

Most models for repeat expansion invoke hairpin loop-outs formed by the individual strands of the expansion-prone repeat tract as intermediates in the expansion process [3–7]. In principle, these loop-outs could form during any time the DNA was unpaired, including during replication, repair synthesis or transcription. Loop-out formation on one strand could lead to the formation of loop-outs on the complementary strand since perfect realignment of the two strands would be blocked. These “double loop-outs” may resemble the Holliday Junctions (HJ) formed during meiosis. We have shown that the FX-repeat loop-outs are bound by MSH2/MSH6 (MutSα) and MSH2/MSH3 (MutSβ), the 2 complexes involved in lesion recognition in mismatch repair (MMR) in mammals [8]. We have also shown that MutSβ is required for almost all expansions in a knock-in FXD mouse model, with MutSα contributing significantly to the MutSβ-dependent expansions [8–12]. Since MutSβ is less much abundant than MutSα in our mouse strain background [8], it suggests that some unique property of MutSβ is important for expansion.

It has been suggested that simple incorporation of the loop-outs could result in expansions [3]. This could occur via a second DNA synthesis step that uses the loop-out as a template. This could involve a non-canonical MMR pathway [13, 14] and some expansion models invoke MutSβ-dependent nick-directed excision of one or both strands as the first step in this process [5, 15–17]. MLH1/PMS2 (MutLα) or MLH1/MLH3 (MutLγ) normally act downstream of the MutS proteins to coordinate excision in MMR. MutSβ binding to repeat-containing loop-outs can trigger MutLα cleavage that can occur on either strand [14]. Such cleavage could provide the nick(s) necessary for excision to take place [13, 14, 18]. Since EXO1 normally acts downstream of the MutL proteins in mismatch excision and is the only exonuclease thus far implicated in MMR, EXO1 may be the protein responsible. However, it is also possible that, instead of such a loop incorporation step, the loop-outs are channeled to a different repair pathway that ultimately leads to expansions.

To address events occurring downstream of MutSβ in the expansion process we decided to test the effect of a null mutation in *Mlh3* in our mouse model since MutLγ interacts preferentially with MutSβ [19, 20]. MutLγ is also known to be required for expansions in a mouse model of Huntington Disease, a CAG-repeat expansion disorder [21]. In contrast, the more abundant MutLα complex either plays a smaller role in expansion in other model systems [16], or is protective [22, 23]. We also tested the effect of an *Exo1* null mutation [24] and a point mutation in the EXO1 nuclease catalytic site. The point mutation prevents EXO1 acting in MMR but does not affect its ability to act in a structural capacity in meiosis where it is required for the proper orientation of cleavage of Holliday Junctions (HJs), a step that involves MutLγ, but not MutLα.

We show here that MutLγ is required for all germ line and somatic expansions in the FXD mouse. However, rather than promote expansion, we found that EXO1 protects against it. It does so in two distinct ways, one that is dependent on its nuclease activity and one that is not. This has interesting implications for the expansion mechanism.

## Results

### MLH3 is essential for somatic expansions

**Fig 1:**
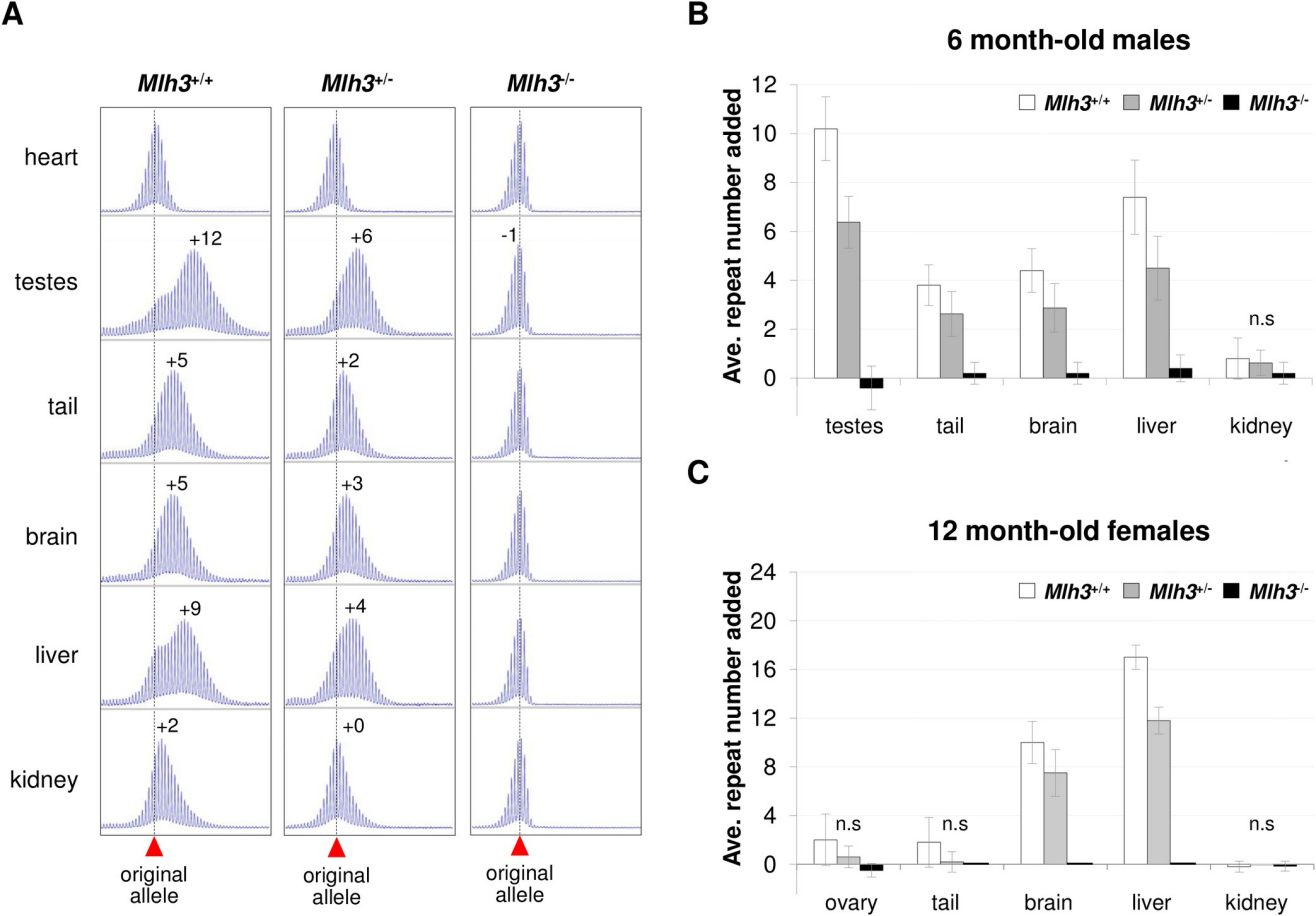
The effect of an *Mlh*3-deficiency on the extent of expansion in different mouse tissues. Statistical analysis of the data shown in this figure is described in the Material and Methods section. A). Representative repeat PCR profiles from the indicated organs of 6-month old *Mlh3*^+/+^, *Mlh3*^+/-^ and *Mlh3*^*-/-*^ male mice with 150–160 repeats. The numerals indicate the number of repeats added to expanded alleles relative to the repeat number seen in heart. The dotted line indicates the size of the original allele. B). The average number of repeats added to the PM allele in the indicated organs of 5 *Mlh*3^+/+^, 8 *Mlh*3^+/-^ and 5 *Mlh*3^-/-^ 6-month-old male mice with 150–160 repeats. The error bars indicate the standard deviations; n.s: not significant. The effect of *Mlh3* dosage was significant (p < 0.0001) for all organs except kidney. The within organ differences for *Mlh3*^+/+^ and *Mlh3*^+/-^ mice were significant for testes (p = 0.002), tail (p = 0.053) and liver (p = 0.008). Differences between *Mlh3*^+/-^ and *Mlh3*^*-/-*^ mice were significant for testes (p = 0.002), tail (p = 0.007), brain (p = 0.014) and liver (p = 0.002). Differences between *Mlh3*^*+/-*^ and *Mlh3*^*-/-*^ were significant for testes (p = 0.008), tail (p = 0.015), brain (p = 0.024) and liver (p = 0.008). C) The average number of repeats added to the PM allele in the indicated organs of 5 *Mlh*3^+/+^, 8 *Mlh*3^+/-^ and 5 *Mlh*3^-/-^ 12 months old female mice with 150–160 repeats. The error bars indicate the standard deviations; n.s: not significant. The effect of *Mlh3* gene dosage in females was significant for ovary (p = 0.008), brain (p = 0.001) and liver (0.0005). In liver all the within organ differences were significant for *Mlh3*^+/+^ and *Mlh3*^+/-^ (p = 0.04), *Mlh3*^+/+^ and *Mlh3*^*-/-*^ (p = 0.02) and *Mlh3*^+/-^ and *Mlh3*^*-/-*^ (p = 0.007) comparisons. In brain, significant differences were only seen for *Mlh3*^+/+^ and *Mlh3*^*-/-*^ (p = 0.024) and *Mlh3*^+/-^ and *Mlh3*^*-/-*^ (p = 0.007) comparisons. No significant differences were seen for ovary, likely because of the very small number of expansions seen even in *Mlh3*^+/+^ animals at this age.

In order to assess the role of *Mlh3* in somatic expansions we compared the repeat PCR profiles in different organs of 6 months-old *Mlh3*^+/+^, *Mlh3*^*+/-*^ and *Mlh3*^*-/-*^ male mice that had inherited alleles with 150–160 repeats and determined the average repeat number added to the expanded alleles as an indicator of the extent of expansion. With the exception of heart, an organ that shows no postnatal expansion, expansion was less extensive in the organs of a *Mlh3*^+/-^ male than in the organs of the *Mlh3*^+/+^ male, while in the *Mlh3*^*-/-*^ male, no evidence of expansion was seen in any of the tissues tested (Fig 1A). When the repeat number added to the expanded allele in each organ from multiple animals was averaged, the effect of the loss of one or both *Mlh3* alleles was found to be highly significant for all organs (Fig 1B; p < 0.0001). The number of repeats added was significantly lower in *Mlh*3^+/-^ males than in *Mlh*3^+/+^ males in all expansion-prone organs and in *Mlh*3^-/-^ males the average number of repeats added was <0.5 repeat for all organs, a result that falls within the margin of error of the assay. Since females show much less extensive expansions than males [25], we examined the effect of the loss of *Mlh3* in females at 12 months of age. Even at this age expansions in some organs are too small for differences between *Mlh3*^+/+^ and *Mlh3*^*-/-*^ mice to reach statistical significance. However, while expansions are clearly seen in the ovary, brain, liver and tail of *Mlh3*^+/+^ females, no expansions were seen in *Mlh3*^*-/-*^ females in any organ (Fig 1C) and the difference between the extent of expansion in the brains and livers of these animals was large enough to be statistically significant. Thus, we conclude that *Mlh*3 is required for all somatic expansions in both males and females.

This effect is not mediated via an effect on the levels of MutLα since, with the possible exception of testis, the loss of MLH3 does not affect the levels of either MLH1 or PMS2, the constituents of the MutLα complex (S1 Fig). In the case of testis, PMS2 levels were elevated, while MLH1 levels were unaltered. This would be consistent with the idea that when MLH3 levels decrease, more MLH1 is available to form a heterodimer with PMS2. This would reduce PMS2 degradation, analogous to what is seen with constituents of the MutS complexes [26–28]. This effect may be limited to testis since MLH3 is normally present at ~60-fold lower levels than MutLα in somatic cells [29].

### MLH3 is also required for germ line expansion

Since *Mlh3*^*-/-*^ mice are sterile because of a defect in crossing over in meiosis [30], we could not monitor the incidence of germ line transmission of expanded alleles in these animals. However, the testis of these animals shows no evidence of expansion (Fig 1A and 1B). We have previously demonstrated that expansions are limited to premeiotic stages of gametogenesis (S2 Fig) [31]. In WT animals a bimodal distribution of repeat sizes is seen in the testis with the smaller peak corresponding to unexpanded alleles and the larger peak to the expanded alleles [31]. A single peak of the same size as the expanded allele seen in testis can be seen in the primary spermatocytes and the size of this allele does not change in more mature gametes [31]. While *Mlh3*^-/-^ male mice do not have secondary spermatocytes or mature sperm, they do have primary spermatocytes [30]. Thus, any expansions in *Mlh3*^*-/-*^ mice should be apparent as a second peak in the testis repeat PCR profile. Since the testis PCR profile lacks a second peak and is, in fact, indistinguishable from the heart PCR profile, we conclude that in addition to being required for somatic expansion, *Mlh*3 is also required for germ line expansion in males. *Mlh3*^+/-^ males also show a significantly smaller number of repeats added to the PM allele in the testis, consistent with fewer repeats having been added to their gametes.

**Fig 2:**
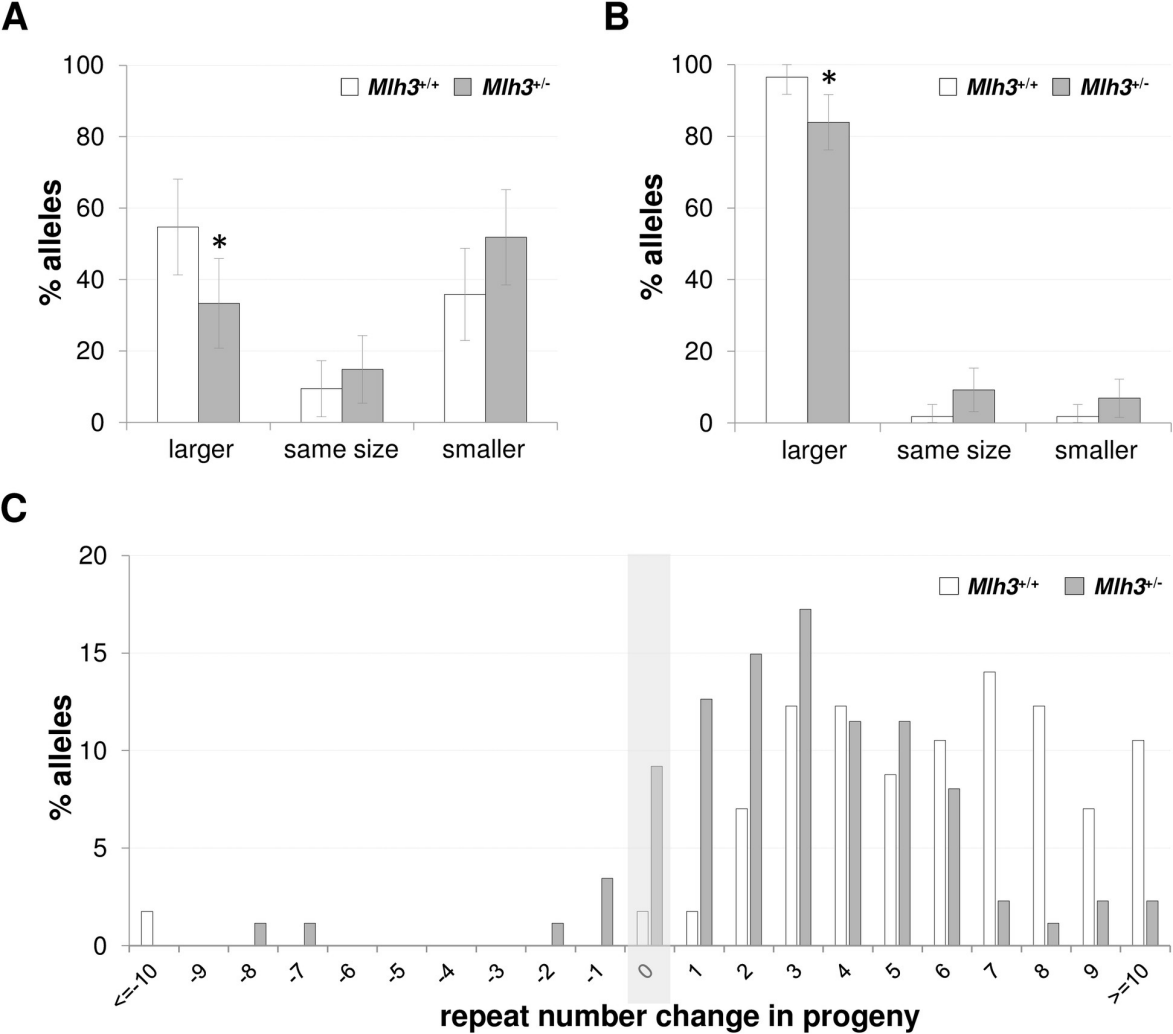
The effect of heterozygosity for *Mlh*3 on expansion in the gametes and intergenerational expansion. Statistical analysis of the data shown in this figure is described in the Material and Methods section. A) The distribution of alleles in individual mature gametes of 3-month old *Mlh*3^+/+^ and *Mlh*3^+/-^ males. The number of alleles that were larger than the parental allele, the same size or smaller than the parental allele was determined based on small pool PCR of the sperm collected from 2 males of each genotype. The error bars represent the 95% confidence intervals. A total of 53 individual sperm from *Mlh*3^+/+^ mice and 54 sperm from *Mlh*3^+/-^ mice with 155–157 repeats were examined (p = 0.03). B) A graphical representation of the number of alleles that were larger, the same size or smaller than the maternal allele for 2–6 months old age-matched females with 145–152 repeats. 87 pups from 6 *Mlh*3^+/-^ and 57 pups from 4 *Mlh*3^+/+^ females were analyzed. The error bars represent the 95% confidence intervals. C) Distribution of the change in the repeat number in the progeny of the mothers shown in panel B. The mean number of repeats added to the PM allele in the progeny of *Mlh3*^+/-^ mice was 3.7 compared to 6.0 in *Mlh3*^+/+^ mice (p < 0.0001).

Small pool PCR from 3-month-old *Mlh3*^+/-^ males shows clearly that they have fewer expanded alleles in their gametes than *Mlh3*^+/+^ males of the same age (Fig 2A). Thus, our data demonstrate that MLH3 is required for all germ line and somatic expansion in male FXD mice and that even heterozygosity for the null allele causes a significant decline in the extent of both germ line and somatic expansions.

The progeny of *Mlh*3^+/-^ mothers showed a small reduction in the proportion of expanded alleles compared to the progeny of age-matched *Mlh3*^+/+^ mothers (Fig 2B). Furthermore, the average size of the expansions in the progeny of *Mlh3*^+/-^ mothers was also significantly smaller than those in the progeny of *Mlh*3^+/+^ mothers (Fig 2C). We have previously shown that expansions continue to accumulate on previously expanded alleles as animals age [12, 31, 32]. Thus, the smaller size of the transmitted alleles from *Mlh3*^+/-^ mothers would be consistent with them having undergone fewer rounds of expansion. Thus, as with males, even the loss of a single functional Mlh3 allele is enough to reduce the extent of germ line expansion in females.

### EXO1 reduces the extent of both intergenerational and somatic expansion

To evaluate a role of EXO1 in expansion we tested the effect of both an *Exo1* null mutation and a D173A mutation. The D173A mutation is located in the active site of EXO1 and thus abolishes its hydrolytic activity. However, X-ray crystallography and *in vitro* biochemistry indicates that the protein retains its native conformation and DNA binding affinity [33–35] and it is expressed at similar levels as WT EXO1 in our mouse strain background (Chahwan et. al., manuscript in preparation). *Exo1* null mice, like *Mlh3*^*-/-*^ mice, are defective in MMR and are sterile because they are unable to complete crossing-over during meiosis and thus make no mature gametes [24]. Mice homozygous for the mutant allele (*Exo1*^A/A^) are MMR defective, but fertile consistent with the idea that *Exo1* plays a structural role in meiosis [36].

**Fig 3:**
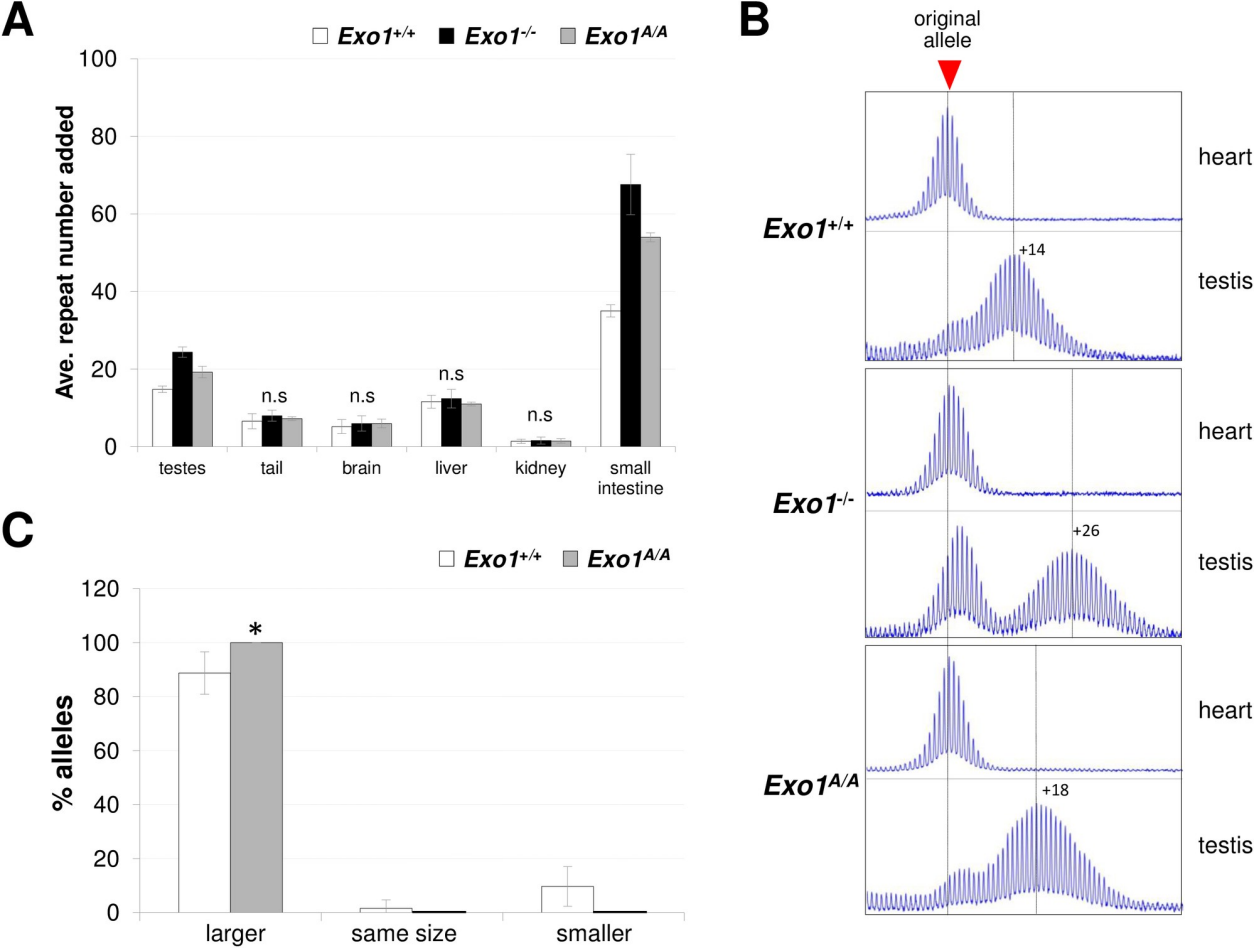
Expansion in different organs of *Exo1*^*+/+*^, *Exo1*^*-/-*^ and *Exo1*^A/A^ male mice. Statistical analysis of the data shown in this figure is described in the Material and Methods section. A) The average number of repeats added to the PM allele in different organs of 5 *Exo1*^+/+^, 5 *Exo1*^*-/-*^ and 4 *Exo1*^A/A^ 6-month-old male mice. The original inherited allele in each case had 172–179 repeats. The repeat number added represents the difference of the repeat size in the indicated organ relative to the repeat number in heart, an organ that shows no expansion and thus which reflects the size of the original inherited allele. The error bars indicate the standard deviation. The effect of genotype was only significant for testis and small intestine (p < 0.0001). In these organs, the within group differences i.e. the differences between expansions in *Exo1*^*+/+*^ and *Exo1*^*-/-*^, *Exo1*^*+/+*^ and *Exo1*^A/A^ and *Exo1*^*-/-*^ and *Exo1*^A/A^ mice were all significant (p = 0.016). B) Representative repeat PCR profiles from the heart and testis of mice with the indicated *Exo1* genotypes. C) Proportion of alleles that were larger than, smaller than and the same size as the parental alleles in the progeny of *Exo1*^*+/+*^ and *Exo1*^A/A^ male mice 62 pups from 7 *Exo1*^*+/+*^ and 35 pups from 6 *Exo1*^A/A^ males were analyzed (p = 0.04).

When we examined the number of repeats added to the PM allele in testes, tail, brain, liver, and kidney, organs we have examined in previous studies [8, 10, 11, 37], large differences between *Exo1*^*+/+*^ and *Exo1*^*-/-*^ male mice was only seen in the testis (Fig 3A and 3B). A failure to see large differences in the tail, kidney, liver and brain of male mice is consistent with the fact that EXO1 not highly expressed outside of the testis [38, 39]. However, it is known that the small intestine shows an increased mutation rate in the absence of EXO1 [40]. We therefore decided to also test this organ for expansions. Alleles in the small intestine of *Exo1*^*-/-*^ mice gained roughly twice as many repeats as the *Exo1*^*+/+*^ mice (Fig 3A and S3 Fig). The *Exo1*^*A/A*^ mice also showed the gain of significantly more repeats than *Exo1*^*+/+*^ mice but, as in testis, the number of repeats gained was fewer than in the *Exo1*^*-/-*^ mice. The differential effect of the null and D173A mutation in small intestine suggests that EXO1 plays both a nuclease-independent and a nuclease-dependent role in reducing somatic expansions in this tissue. The failure to see large changes in other somatic tissue of males may reflect the relatively low level of expression of EXO1 in these tissues. In WT females, the somatic expansion frequency is much lower than it is in males [25, 37]. This makes it difficult to accurately determine the mean expansion size or the somatic instability index. Expansions in different females are also much more variable, due in part to the fact that expansion only occurs when the repeat is on the active X chromosome [25]. Since X chromosome inactivation is a stochastic process, female mice show a wide variation in the fraction of expanded alleles that are on the active X [25]. However, while direct comparisons are difficult, expansions, in general, do seem to be more extensive in *Exo1*^*-/-*^ and *Exo1*^A/A^ females than in *Exo1*^*+/+*^ females (S4 Fig, panel B).

The testes of *Exo1*^*-/-*^ mice lack mature gametes that make up ~95% of the testicular cells. They thus produce a repeat PCR profile that differs from what is seen in *Exo1*^*+/+*^ mice and *Exo1*^A/A^ mice (Fig 3B). The presence of a peak corresponding to the original allele size reflects the fact that the somatic cells of the testes [41], where the repeat does not expand, constitutes a greater fraction of the testicular cells in *Exo1*^*-/-*^ mice than they do in *Exo1*^*+/+*^ and *Exo1*^A/A^ mice. Nevertheless, it is clear that the residual gametes showed an average gain of 24 repeats in *Exo1*^*-/-*^ mice compared to 14 repeats in *Exo1*^*+/+*^ mice and thus that expansion in the gametes was more extensive in *Exo1*^*-/-*^ mice. This was consistent with the gain of repeats seen in purified primary spermatocytes purified from *Exo1*^*-/-*^ mice (S2 Fig). As in the small intestine, *Exo1*^A/A^ mice had gained considerably more repeats in the testis than the *Exo1*^*+/+*^ mice, but significantly fewer repeats than the *Exo1*^*-/-*^ mice. This data suggests that, as with somatic expansions, EXO1 protects against germ line expansions in both a nuclease-dependent and nuclease-independent manner.

**Fig 4:**
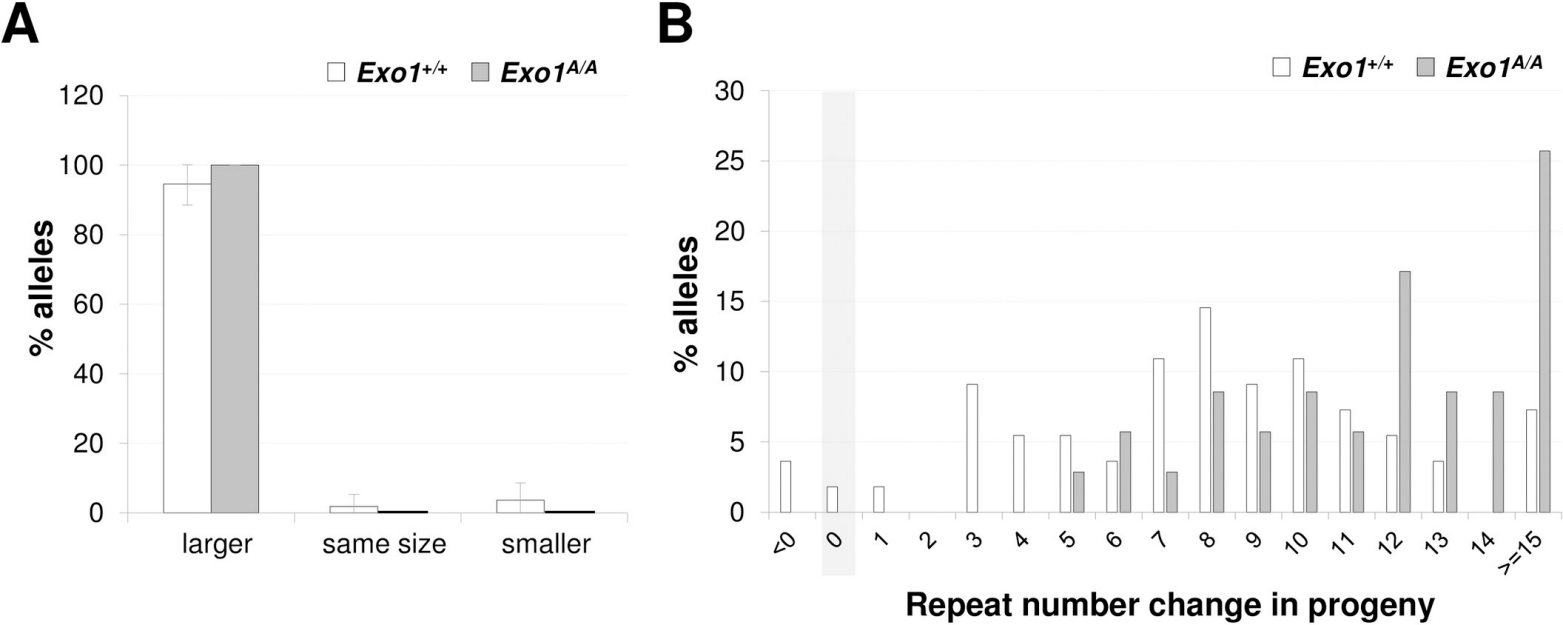
Maternal transmission of the PM allele in *Exo1*^*+/+*^ and *Exo1*^A/A^ mice. Statistical analysis of the data shown in this figure is described in the Material and Methods section. A) Proportion of alleles that were larger, smaller or the same size as the parental alleles in the progeny of 2–6 months old *Exo1*^*+/+*^ and *Exo1*^A/A^ females with 165–175 repeats. 55 pups from 7 *Exo1*^*+/+*^ and 35 pups from 5 *Exo1*^A/A^ females were analyzed. B) Distribution of the change in the repeat number in the progeny of the mothers shown in panel A. The mean number of repeats added to the PM allele in the progeny of *Exo1*^*A/A*^ mice was 12.5 compared to 9.3 in the progeny of *Exo1*^*+/+*^ mice (p < 0.0001).

We verified the effect of losing EXO1 exonuclease activity on germ line expansion by comparing the proportion of expanded alleles transmitted from *Exo1*^A/A^ sires and dams. Consistent with our interpretation of the data from testes, *Exo1*^A/A^ males had significantly more progeny with expansions than *Exo1*^*+/+*^ mice (Fig 3C). No significant differences in the proportion of expanded alleles were seen on maternal transmission (Fig 4A). This is likely because the fraction of expanded alleles was already so high in this population. However, the progeny of *Exo1*^A/A^ mothers had significantly larger alleles than the progeny of *Exo1*^*+/+*^ mothers (Fig 4B). This is consistent with our previous demonstration that expansions continue to accumulate with time on previously expanded alleles [31] and suggests that the loss of EXO1’s nuclease activity alone is sufficient to significantly increase expansions. Failure to see an effect of EXO1 in ovary may reflect the fact that oocytes represent a relatively small fraction of the cells of this organ making any specific effect in the gamete difficult to discern. Nonetheless, our data supports the contention that EXO1 protects the genome against intergenerational repeat expansion in both males and females.

## Discussion

We have shown that MLH3, a component of the MutL complex, MutLγ, is required for both somatic and germ line expansions in both males and females in the FXD mouse model (Figs 1 and 2). The effect of the loss of MutLγ on germ line expansion has not previously been reported, although a similar dependence on MutLγ for somatic expansions was seen in a mouse model of Huntington Disease [21] and a cell culture model of GAA-repeat expansion [22]. The role of MutLγ, coupled with the requirement for MutSβ, increases the likelihood that a similar basic mechanism accounts for all expansions in these disorders, despite differences with respect to which tissues are expansion prone and the contribution of MutSα to expansions in the FXD mouse model [8] and in FRDA iPSCs [42], but not in models of other Repeat Expansion Disorders [43–45]. We have also shown that not only is EXO1 not required for expansion, but it is actually protective, reducing the extent of both germ line and somatic expansions (Figs 3 and 4). We also showed that *Exo1*^A/A^ animals have significantly more expansions than *Exo1*^*+/+*^ mice, but significantly fewer expansions than *Exo1*^*-/-*^ mice. Given that the available data suggest that the D173A protein has a similar overall structure [33–35] and stability as the WT protein (Chahwan et al., manuscript in preparation), our data are consistent with the hypothesis that EXO1 reduces expansion in at least two different ways, one that is dependent on its nuclease activity and one that is not.

The fact that EXO1 is not required for expansions, but is actually protective, suggests that models that propose a role for excision of the strand opposite loopouts by enzymes like EXO1 (e.g., [5, 15–17]), may not account for expansions in the FXD mouse. Furthermore, while it is formally possible that EXO1 provides protection against expansions that occur via a MutS/MutLγ-independent pathway that is not seen when EXO1 is present, the most parsimonious explanation of our data is that EXO1 is acting as it usually does downstream of the MutS/MutL proteins, where it may be functioning, at least in part, to repair the repeat tract in a manner consistent with its normal role in MMR. Experiments are underway to test this hypothesis.

Since EXO1 is protective, it may be that expansions arise via the *Exo1*-independent sub-pathway of MMR [46, 47]. This pathway has been suggested to involve strand displacement or excision by one or more other nucleases [46]. It has been suggested that Artemis, FAN1 and/or MRE11 may be the nucleases involved [47]. However, since EXO1 is protective, it is unclear how these other nucleases would act to promote expansions and in fact, we have recently demonstrated that FAN1 is also protective [41]. Interestingly, the protective effect of FAN1 and EXO1 seem to be complementary, with EXO1’s effect being most apparent in the gonads (this manuscript) and FAN1’s effect being strongest in somatic tissue [41].

While our data shows that expansion proceeds via an EXO1-independent pathway, the fact that EXO1 can protect against expansion in a nuclease-independent way provides an important clue as to the events downstream of MutLγ-binding in the expansion process. MutLγ is much less abundant than MutLα in mammalian cells and unlike MutLα, MutLγ only plays a minor role in MMR. However, MutLγ is essential for processing of Holliday Junctions (HJ) during meiosis, a process in which MutLα plays no role. Thus, MutLγ processing of an intermediate that resembles a HJ may account for the specific requirement for MutLγ in expansions. EXO1 plays an important structural role in facilitating the proper orientation of MutLγ cleavage of HJs during meiosis [36]. We speculate that MutLγ processing of a HJ-like intermediate in the absence of EXO1 would generate staggered double strand breaks that could then be processed to generate expansions. A simple model for such a process is illustrated in S4 Fig. In the presence of EXO1, MutLγ processing may result in products that are processed in a way that does not generate expansions.

Human Genome-Wide Association Studies (GWAS) show that genetic factors previously implicated in mouse models of the Repeat Expansion Diseases, including MSH3 and FAN1, are important disease modifiers for a variety of these disorders [48–50]. This would suggest that expansions in mice and humans share a common mechanism. In light of that, our data demonstrating that EXO1, like FAN1, protects against expansions, suggests that EXO1 variants may also be important modifiers of disease risk.

MutLγ has been implicated in chromosome breakage/fragility of CAG-repeats in yeast [51]. This is consistent with a role of MutLγ in the generation of double-strand breaks, a role we suggest MutLγ plays in repeat expansion in the FXD mouse. Mutations in the putative endonuclease domain of Mlh3 results in meiotic defects [52] and the preferential knockdown of the Mlh3 isoform that contains the nuclease domain reduces expansion in a tissue culture model of GAA-repeat expansion [22]. Thus, the genetic data support a nucleolytic role for MutLγ in repeat expansion. However, no specific MutLγ cleavage has, as yet, been demonstrated on synthetic substrates [19]. Thus, further work is needed in order to be able to test this model and to better understand the events responsible for repeat expansion.

## Materials and methods

### Reagents and services

All reagents were from Sigma-Aldrich (St. Louis, MO) unless otherwise specified. Primers were from Life Technologies (Grand Island, NY). Capillary electrophoresis of fluorescently labeled PCR products was carried out by the Roy J Carver Biotechnology Center, University of Illinois (Urbana, IL).

### Mouse generation, breeding and maintenance

The generation of the FXD mice was described previously [53]. The mice with null mutations in *Mlh3* were obtained from Paula Cohen (Cornell University, Ithaca, NY) [30]. The generation of *Exo1*^*-/-*^ mice has been previously described [24]. A full description of the generation of *Exo1*^A/A^ mice will be published elsewhere (Chahwan et al, manuscript in preparation). Briefly, the mice were generated by CRISPR/Cas9 mediated gene editing of C57BL/6 zygotes with a guide RNA targeting the area containing *Exo1* codon D173 in exon 6 (5’-ACUCUGACCUCCUCGCAUUUGG-3’) and donor DNA carrying the desired mutation and 1.0 kb homologous arms on each side (illustrated in S5 Fig). The resulting offspring were genotyped by PCR and Sanger sequencing to identify founders carrying the EXO1 D173A mutation (S5 Fig). The founders (F0) were mated with wild type C57BL6 mice to produce F1 heterozygotes carrying the D173A mutation. F1 mice were backcrossed to C57B6 mice (4-6x). The mutant protein was expressed a level similar to wild type EXO1 protein (Chahwan et al, manuscript in preparation). All mice were on a C57BL/6 background. Mice were maintained in accordance with the guidelines of the NIDDK Animal Care and Use Committee, who approved this research (ASP-K021-LMCB-15) and in accordance with the Guide for the Care and Use of Laboratory Animals (NIH publication no. 85–23, revised 1996). Euthanasia was carried out using compressed CO2 followed by cervical dislocation.

### DNA isolation

DNA from mouse tails at 3-week-old for genotyping was extracted using KAPA Mouse Genotyping Kit (KAPA Biosystems, Wilmington, MA). DNA from sperm was extracted as previous described [10, 41]. Briefly, sperm were collected by centrifugation at 500 g for 5 minutes then resuspended in 300 μL ATL buffer (Qiagen, Hilden, Germany) containing 0.55 mg/mL Proteinase K and 30 mM DTT and incubated overnight at 55°C. The samples were then mixed with 90 μL of 5 M NaCl and centrifuged at 13,000 g for 10 minutes. The supernatant was transferred to a new tube, mixed with 390 μL ethanol and placed at −20°C for 1 hour. The DNA was then pelleted by centrifugation at 13,000 g for 10 minutes, washed with 70% ethanol and dissolved in TE buffer at 55°C for 15 minutes. DNA was isolated from the organs of 6-month old male mice using a Maxwell 16 Mouse Tail DNA Purification kit (Promega, Madison, WI) according to the manufacturer’s instructions. A 5 cm region of the jejunum starting 10 cm downstream of stomach was used as the small intestinal sample and the DNA was isolated from this segment as described above for intact organs.

### Genotyping and analysis of repeat number

Genotyping of *Mlh3* and *Exo1* null mice was carried out with KAPA mouse genotyping kit (KAPA Biosystems, Wilmington, MA) according to the manufacturer’s instructions with primers Mlh3A (also known as Primer 12265) (5’-GGCCTCTTCGCTATTACGC-3’)/Mlh3B (Primer 16984) (5’-AAGCCAGTGTCTGCCACTCC-3’) primer pair to detect the mutant *Mlh3* allele and Mlh3B/Mlh3C (Primer 16985) (5’-CCCACCTTCTCTACATCGTC-3’) to detect the WT *Mlh3* allele (as described at https://www2.jax.org/protocolsdb/f?p=116:5:0::NO:5:P5_MASTER_PROTOCOL_ID,P5_JRS_CODE:24295,018845). The Exo1A (5’-CTCTTGTCTGGGCTGATATGC-3’)/Exo1B (5’-ATGGCGTGCGTGATGTTGATA-3’) primer pair was used to detect the WT *Exo1* allele and Exo1C (5’-AGGAGTAGAAGTGGCGCGAAGG-3’)/Exo1B to detect the mutant *Exo1* null allele. *Exo1* D173A genotyping was carried out using an Amplification‐Refractory Mutation System (ARMS)-based assay [54] that we designed (illustrated in S5 Fig). Briefly, the tetra-primer pairs for this assay were designed using BatchPrimer3 (https://wheat.pw.usda.gov/demos/BatchPrimer3/). The primers Exo1-ARMS-AF1 (5’-GAAATGGCTTTTGGAAAGTTTTGTTCGC-3’)/Exo1-ARMS-AR2 (5’-CTTCTTACAGCCAAATGCGAGGAAG**G**-3’) were using for the mutated *Exo1* A (GCC) allele and the primers Exo1-ARMS-DF2 (5’-CAGGCTGTCATCACAGAGGACTCCG**A**-3’)/ Exo1-ARMS-DR1 (5’-CCAAACTCCAAAGGATAAAACCAAGCCC-3’) were using for the WT *Exo1* D (GAC) allele. The primer bases shown in bold are allele specific. The underlined bases in the primer are mismatches introduced to improve the specificity of the assay. The PCR mix contained 10 ng DNA, 1x PCR buffer, 0.2 mM dNTPs, 0.5 μM of each primer and 1 U JumpStart REDTaq, and the PCR parameters were 1x 96°C for 3 minutes, 8x (94°C for 30 seconds, 72–65°C (-1°C/cycle) for 30 seconds, 72°C for 1 minutes), followed by 27x (94°C for 30 seconds, 65°C for 30 seconds, 72°C for 1 minutes), and ending with 72°C for 10 minutes. *Fmr1* PM allele genotyping and repeat size analysis was carried out using a fluorescent PCR assay and FAM-labelled FraxM4 (FAM-5’-CTTGAGGCCCAGCCGCCGTCGGCC-3’) and FraxM5 (5’-CGGGGGGCGTGCGGTAACGGCCCAA-3’) primer pair as described previously [31]. Small pool PCR was used to analyze sperm DNA as previously described [10]. The PCR reactions were resolved by capillary electrophoresis on an ABI Genetic Analyzer [37]. The resultant fsa file was then displayed using a custom R script [55] that is available on request. For intergenerational (germ line) transmissions the number of alleles that were larger, smaller or the same size as the parental allele were then scored.

The Somatic Instability Index (SII) typically used to quantify somatic expansions [57] is sensitive to the ratio of cells containing expanded alleles to the cells lacking expanded alleles. Therefore, it is not suitable for comparing somatic expansions in the testes of wildtype and either *Mlh3*^-/-^ or *Exo1*^-/-^ mice, since the *Mlh3*^*-/-*^ and *Exo1*^*-/-*^ mice lack mature gametes that constitute the bulk of cells present in the wildtype testes. It is also unreliable in organs like small intestine, where cells of the mucosal layer, which show a high degree of expansions, are easily lost during isolation. We therefore used slight modification of a previously described approach to quantitate somatic expansions [56]. We have previously shown that heart shows no postnatal expansions [37]. The peak seen in the repeat PCR profile from heart thus reflects the original inherited allele size. The PCR profiles for other organs show either a single peak that is larger than the peak seen in heart or two peaks, one corresponding to the original allele and the second corresponding to the expanded allele. The size of the expanded allele and the original allele are obtained from the repeat PCR profiles and the difference between them divided by 3 to obtain the repeat number added to the expanded allele. This measure correlates well with the SII for tissues where the SII is appropriate to use.

The extent of expansion in different tissues was compared in animals of 3 different genotypes using a Jonckheere-Terpstra test with permutation-based exact inference and a Hommel procedure to adjust for multiplicity. Pairwise comparisons of organ-tissue and genotype combinations whose differences remained significant at a nominal level (10%) were then carried out using Mann-Whitney U tests (exact inferences), again with a Hommel procedure to adjust for multiple comparisons. These calculations were carried out using R version 3.2 (exactci, COMPoissonReg packages), SAS version 9.4, and StatXact version 8 (cran.r-project.org; www.sas.com; www.cytel.com). Fisher’s exact test for the comparison of the number of intergenerational expansions relative to alleles that did not expand were carried out using the GraphPad QuickCalcs website (http://www.graphpad.com/quickcalcs). Mann-Whitney U tests for comparisons of two sample groups using normal-approximation inferences were carried out using the Vassarstats website (vassarstats.net).

## Supporting information

S1 Fig Levels of MLH1 and PMS2 in *Mlh3*^*-/-*^ animals. Western blots of protein extracts from 6-month old *Mlh3*^+/+^ and *Mlh3*^*-/-*^ mice with MLH1, PMS2 and β-actin antibodies, showing that with the exception of testis, loss of MLH3 does not affect the levels of either MLH1 or PMS2. H: heart, T: testis, B: brain, L: liver and K: kidney.
(TIF)

S2 Fig Expansions are already present in primary spermatocytes of the FXD mouse. Testes cells were purified from *Exo1^+/+^* and *Exo1*^*-/-*^ mice by flow cytometry as described in the Materials and Methods. DNA was then extracted from the indicated cell types along with the heart and the contralateral testis. The Repeat PCR profiles were then determined for the indicated organs and cell types.
(TIF)

S3 Fig Representative repeat PCR profiles from the heart and small intestine of *Exo1^+/+^*, *Exo1*^*-/-*^ and *Exo1*^A/A^ mice. A) Representative repeat PCR profiles in 6-month old male mice. The original inherited allele in each case had 172–179 repeats. The repeat number added to the expanded allele in the small intestine of each mouse is indicated on the scan. The left-hand side of Panel A shows the PCR profile generated using the same flanking primers as used in the data shown in Fig 3B (FraxM4/FraxM5). Because the expansions are so extensive in the small intestine in males we verified the average number of repeats added using a more distal primer pair (Not_mFraxC/Not_FraxR4). As can be seen on the right-hand side of Panel A, the additional flanking bases results in a larger fragment that gives a more compact PCR profile. B) Representative repeat PCR profiles of 6-month old *Exo1*^*+/+*^, *Exo1*^*-/-*^ and *Exo1*^A/A^ females, showing 3 different examples of each genotype. The original inherited allele in each case had 167–174 repeats.
(TIF)

S4 Fig Model for the generation of expansions. Strand-slippage by Polβ results in a loop-out forming on the nascent strand. Since the loop-out is within the repeat, priming from the slipped position may be inefficient. This would favor the formation of a loop-out on the complementary strand to generate a cruciform or HJ-like intermediate. In the absence of EXO1, MutLγ may process the intermediate in the direction indicated by the red arrows. Subsequent melting of the loop-out would allow annealing of the cleaved strands via hydrogen bonding of the resulting 3’ overhangs. If this annealing occurs slightly out of register, the resultant gaps could be filled in to generate small expansions. In the presence of EXO1, cleavage may occur in the orientation indicated by the blue arrows. After loop-out resolution, this would generate 5’ overhangs that could be further processed by EXO1 or other 5’ to 3’ exonucleases. This processing would reduce the length of the 5’ overhang, eliminate it or generate 3’ overhangs. The net effect would be fewer expansions. While expansion is depicted here as being triggered by oxidative damage, in principle the double-loop outs also could form directly any time that the DNA was unpaired.
(TIF)

S5 Fig Verification and genotyping of the *Exo1* D173A mutant mice. A) Schematic representation of CRISPR/Cas9 editing strategy used to generate Exo1 D173A mice. B) Sequencing of the PCR products resulting from amplification across the mutated region in *Exo1*^D/D^, *Exo1*^D/A^ and *Exo1*^A/A^ mice. C) Graphic representation of PCR genotyping assay for *Exo1*^D/D^, *Exo1*^D/A^ and *Exo1*^A/A^ mice. AF1 and DR1 are flanking primers that amplify both the WT and mutant alleles. The DF2 primer has an A at its 3’ end and thus only primes on the WT allele. AR2 contains a C at its 3’ end and thus only primes on the mutant allele. C) Examples of results of PCR genotyping assay for the identification of *Exo1*^D/D^, *Exo1*^D/A^ and *Exo1*^A/A^ mice using all 4 primers as wells the D allele and A allele primer pairs individually.
(TIF)

S1 Supplemental methods (DOCX)
