# Supplementary figures and images for: MutLγ promotes repeat expansion in a Fragile X mouse model while EXO1 is protective
Source: PLoS Genet. 2018 Oct 12;14(10):e1007719. doi: 10.1371/journal.pgen.1007719 (PMC6200270; doi:10.1371/journal.pgen.1007719)

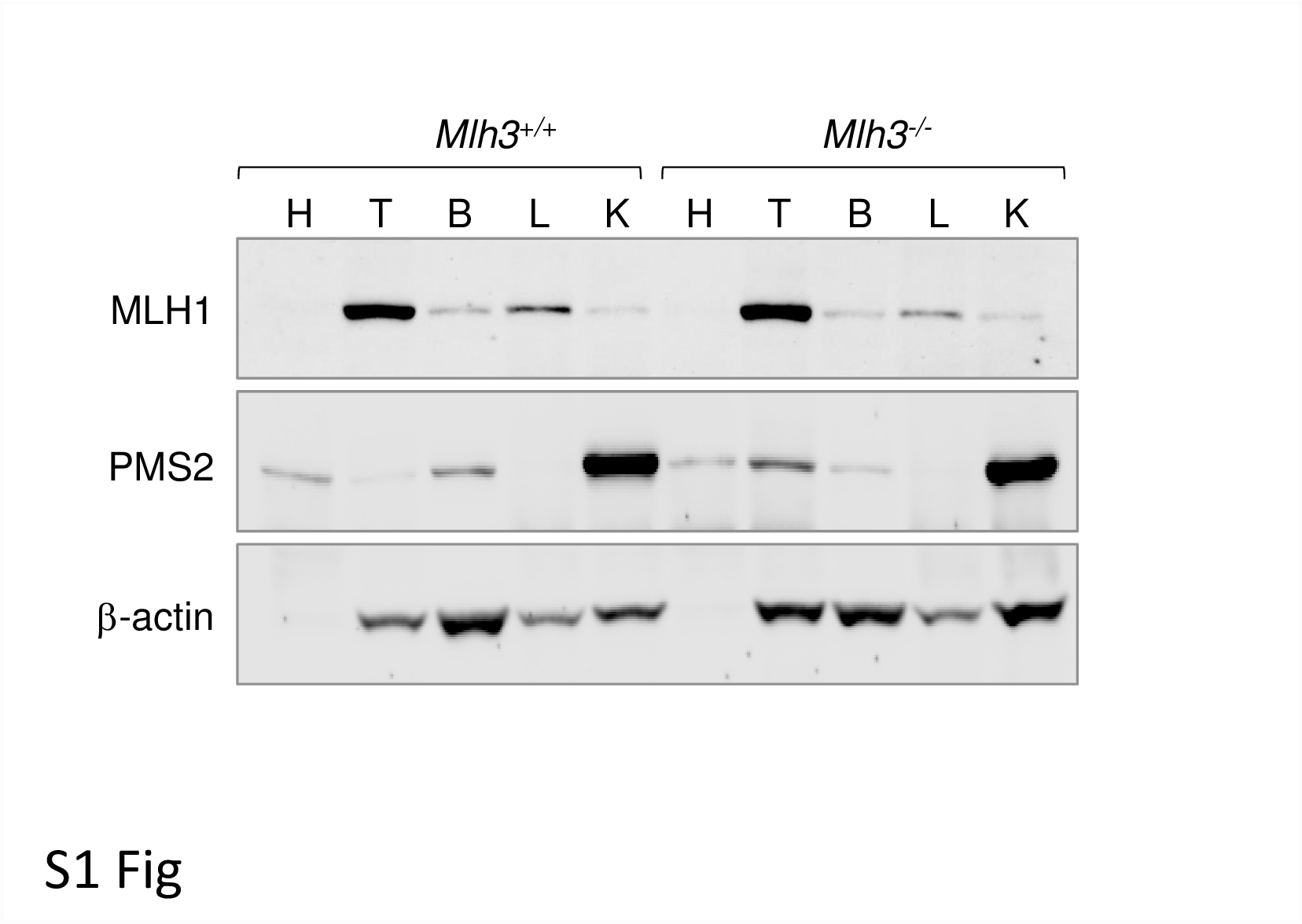

Supplement: S1 Fig — Western blots of protein extracts from 6-month old Mlh3+/+ and Mlh3-/- mice with MLH1, PMS2 and β-actin antibodies, showing that with the exception of testis, loss of MLH3 does not affect the levels of either MLH1 or PMS2. H: heart, T: testis, B: brain, L: liver and K: kidney. (TIF) [file pgen.1007719.s001.tif]

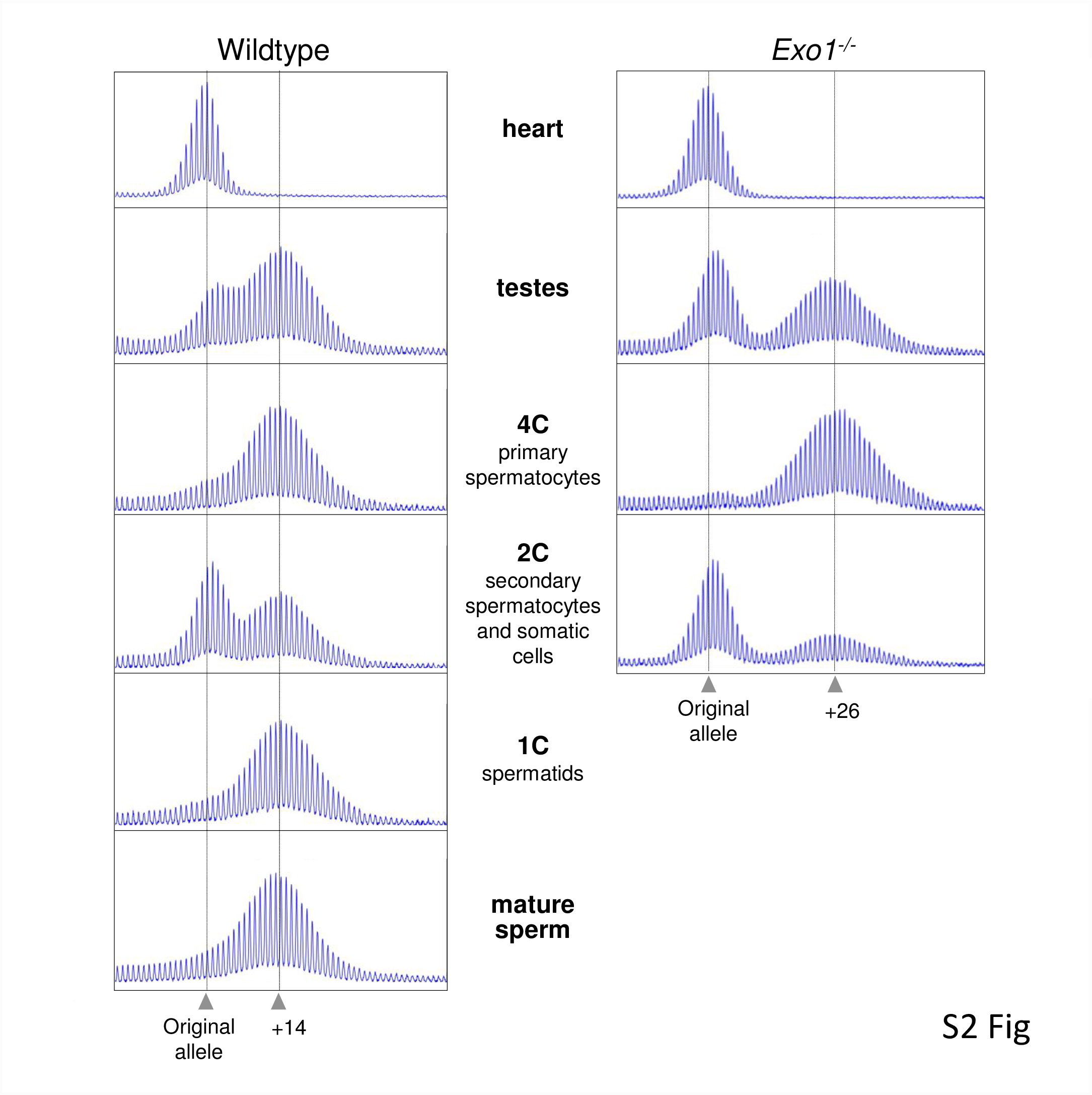

Supplement: S2 Fig — Testes cells were purified from Exo1+/+ and Exo1-/- mice by flow cytometry as described in the Materials and Methods. DNA was then extracted from the indicated cell types along with the heart and the contralateral testis. The Repeat PCR profiles were then determined for the indicated organs and cell types. (TIF) [file pgen.1007719.s002.tif]

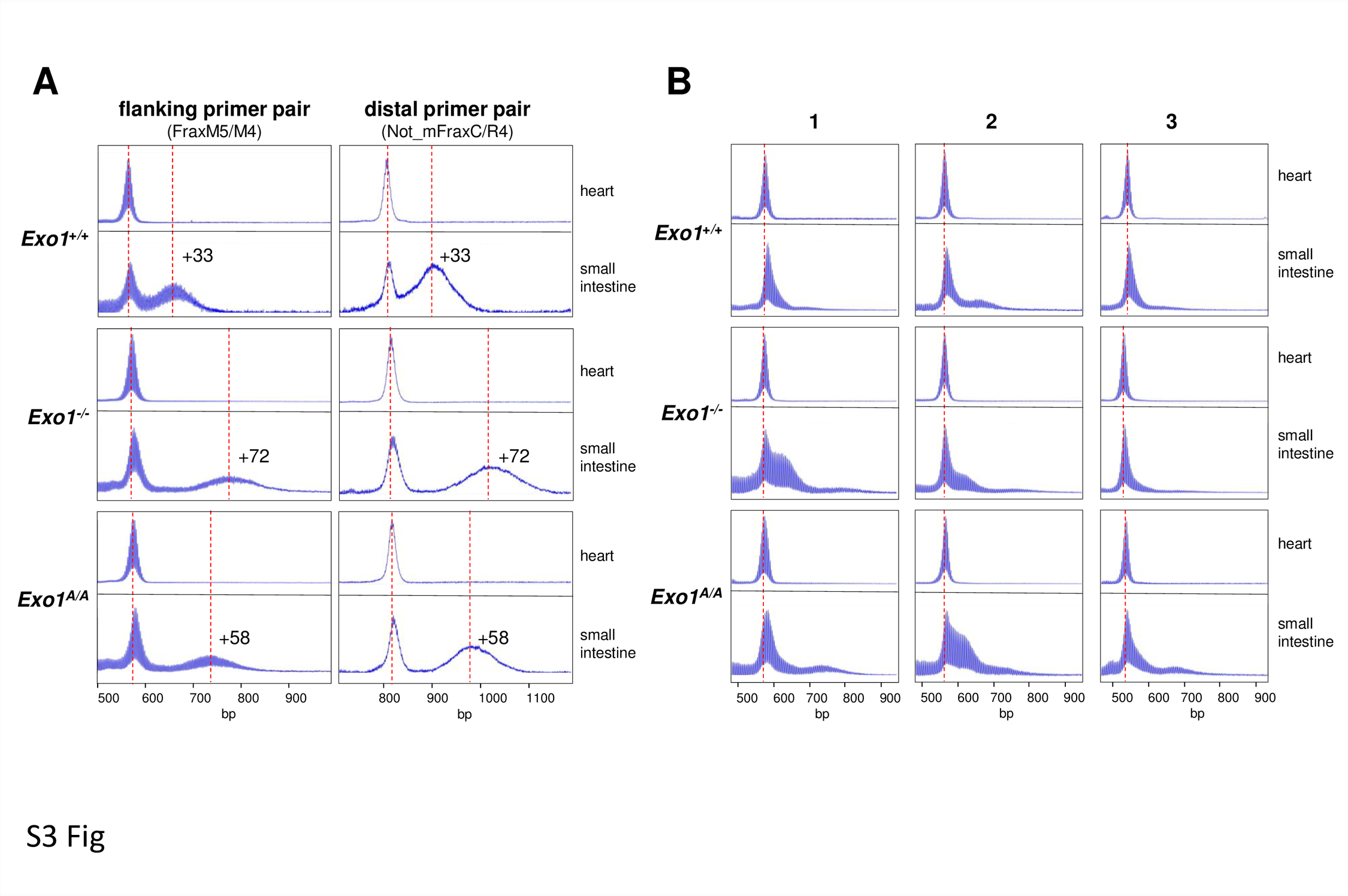

Supplement: S3 Fig — A) Representative repeat PCR profiles in 6-month old male mice. The original inherited allele in each case had 172–179 repeats. The repeat number added to the expanded allele in the small intestine of each mouse is indicated on the scan. The left-hand side of Panel A shows the PCR profile generated using the same flanking primers as used in the data shown in Fig 3B (FraxM4/FraxM5). Because the expansions are so extensive in the small intestine in males we verified the average number of repeats added using a more distal primer pair (Not_mFraxC/Not_FraxR4). As can be seen on the right-hand side of Panel A, the additional flanking bases results in a larger fragment that gives a more compact PCR profile. B) Representative repeat PCR profiles of 6-month old Exo1+/+, Exo1-/- and Exo1A/A females, showing 3 different examples of each genotype. The original inherited allele in each case had 167–174 repeats. (TIF) [file pgen.1007719.s003.tif]

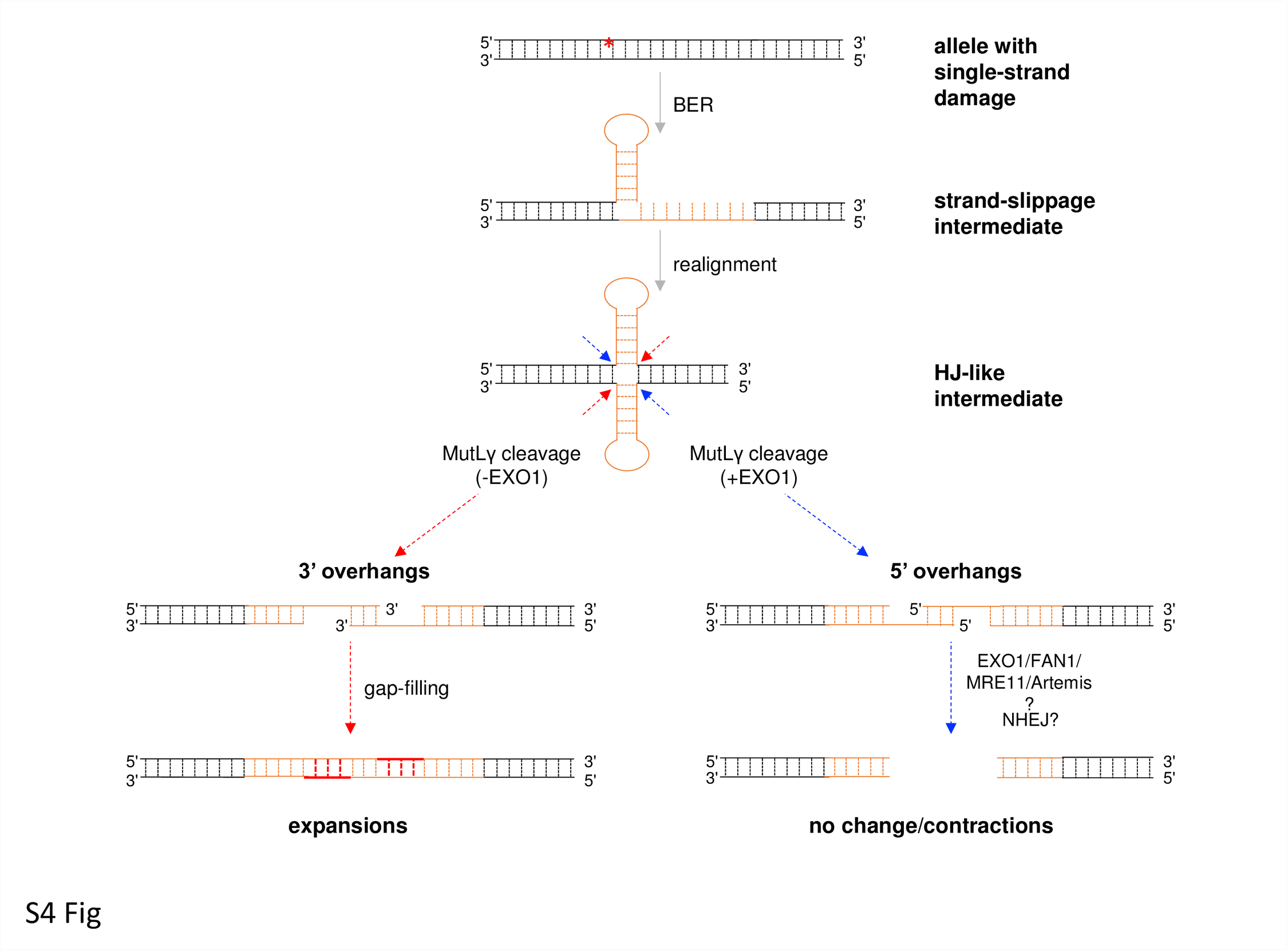

Supplement: S4 Fig — Strand-slippage by Polβ results in a loop-out forming on the nascent strand. Since the loop-out is within the repeat, priming from the slipped position may be inefficient. This would favor the formation of a loop-out on the complementary strand to generate a cruciform or HJ-like intermediate. In the absence of EXO1, MutLγ may process the intermediate in the direction indicated by the red arrows. Subsequent melting of the loop-out would allow annealing of the cleaved strands via hydrogen bonding of the resulting 3’ overhangs. If this annealing occurs slightly out of register, the resultant gaps could be filled in to generate small expansions. In the presence of EXO1, cleavage may occur in the orientation indicated by the blue arrows. After loop-out resolution, this would generate 5’ overhangs that could be further processed by EXO1 or other 5’ to 3’ exonucleases. This processing would reduce the length of the 5’ overhang, eliminate it or generate 3’ overhangs. The net effect would be fewer expansions. While expansion is depicted here as being triggered by oxidative damage, in principle the double-loop outs also could form directly any time that the DNA was unpaired. (TIF) [file pgen.1007719.s004.tif]

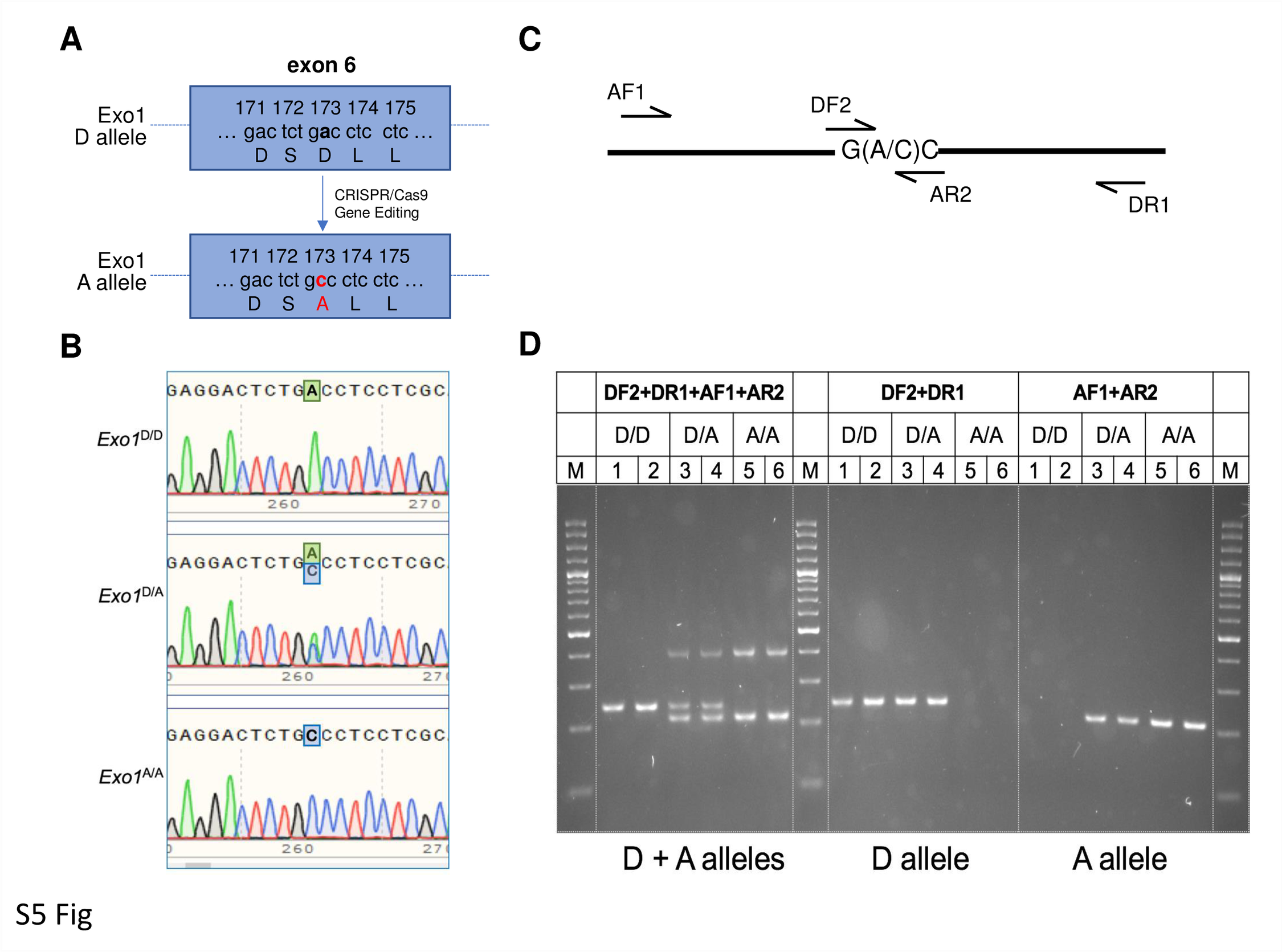

Supplement: S5 Fig — A) Schematic representation of CRISPR/Cas9 editing strategy used to generate Exo1 D173A mice. B) Sequencing of the PCR products resulting from amplification across the mutated region in Exo1D/D, Exo1D/A and Exo1A/A mice. C) Graphic representation of PCR genotyping assay for Exo1D/D, Exo1D/A and Exo1A/A mice. AF1 and DR1 are flanking primers that amplify both the WT and mutant alleles. The DF2 primer has an A at its 3’ end and thus only primes on the WT allele. AR2 contains a C at its 3’ end and thus only primes on the mutant allele. C) Examples of results of PCR genotyping assay for the identification of Exo1D/D, Exo1D/A and Exo1A/A mice using all 4 primers as wells the D allele and A allele primer pairs individually. (TIF) [file pgen.1007719.s005.tif]
