# Supplementary material for: MutLγ promotes repeat expansion in a Fragile X mouse model while EXO1 is protective
Source: PLoS Genet. 2018 Oct 12;14(10):e1007719. doi: 10.1371/journal.pgen.1007719 (PMC6200270; doi:10.1371/journal.pgen.1007719)
Supplement: S1 Supplemental methods — (DOCX) [file pgen.1007719.s006.docx]

**S1 Supplemental Methods**

**Primers**

FraxM4: 5’-FAM-CTTGAGGCCCAGCCGCCGTCGGCC-3

FraxM5: 5’-CGGGGGGCGTGCGGTAACGGCCCAA-3’

Not_mFraxC: 5’-agttcagcggccgcgctggggagcgtttcggtttcacttccggt-3’

Not_FraxR4: 5’-FAM-caagtcgcggccgccttgtagaaagcgccattggagccccgca-3’

**Testicular cell isolation**

Different cell types from the testes were isolated by flow cytometry using a slight modification of a previously published procedure [1]. Briefly, after euthanization the testes were isolated and the tunica albuginea removed. The testes were then macerated and treated with a solution containing 1 mg/mL Collagenase IV (ThermoFisher, Waltham, MA) and 100 U/mL DNase at 37˚C for 20 minutes to release the seminiferous tubules. The seminiferous tubules were wash by PBS three times and collected by centrifugation at 200 g for 5 minutes, followed by digestion with 1 U/mL Dispase (STEMCELL technologies, Vancouver, Canada), and 100 U/mL DNase for 20 minutes at 37˚C. The testicular cells were passed through a 70-μm cell strainer and collected by centrifugation at 500 g for 5 minutes. Cells were then resuspended in PBS with 5% FBS, counted and diluted to 1x10^6^/mL and stained with 5 mg/mL Hoechst 33342 in PBS with 5% FBS at 37˚C for 45 minutes. Cells were then washed with PBS and resuspended in PBS with 5% FBS. A FACS Aria II (BD Biosciences, San Jose, CA) flow sorter was used for sorting. Hoechst fluorescence was detected with a 450-nm band-pass filter for blue fluorescence or a 675-nm band-pass filter for red fluorescence. Compared with WT mice, *Exo1^-/-^* males lack the 1C population that corresponds to the post-meiotic gametes. Cells with 4C, 2C, and 1C DNA content were sorted for blue versus red (450-nm vs. 675-nm band-pass emission, respectively), and collected in PBS with 5% FBS.

**Western blotting**

Total protein extracts were prepared from flash frozen heart, testes, brain, liver, and kidney of 6-month old mice. Tissues were homogenized using a tissue homogenizer (Precellys 24, Bertin Technologies, Berlin, Germany) with T-PER protein extraction reagent (Pierce Biotechnology, Inc., Rockford, IL) supplemented with complete, Mini, EDTA-free protease inhibitor cocktail (Roche Applied Science, Indianapolis, IN). The protein concentrations were determined using a Bio-Rad protein assay kit (Bio-Rad, Hercules, CA) by DU 730 UV/Vis Spectrophotometer (Backman Coulter, Inc., Brea, CA). Proteins were heated for 10 minutes at 70°C in NuPAGE LDS-Sample Buffer (Life Technologies, Grand Island, NY) with NuPAGE sample reducing Agent (Life Technologies), resolved by electrophoresis on either 3–8% NuPAGE Tris-Acetate gels (Life Technologies) and transferred to nitrocellulose membranes using the Trans-Blot Turbo Transfer System (Bio-Rad, Hercules, CA) according to the manufacturer’s instructions. Membranes were blocked for one hour at room temperature in 5% ECL Prime blocking agent (GE Healthcare Bio-Sciences) in TBST, then incubated overnight at 4°C with antibodies to MLH1 (ab92312, Abcam, Cambridge, MA) at a concentration of (1:5000), and PMS2 (sc-25315, Santa Cruz, Dallas, TX) at a concentration of (1:1000). The secondary antibodies (anti-rabbit IgG, NA934V, and anti-mouse IgG, NA931V GE Healthcare) were both used at a dilution of 1:5000. After addition of the ECL Prime detection reagent (GE Healthcare Bio-Sciences), the blot was imaged using ChemiDoc Imaging System (Bio-Rad, Hercules, CA). Beta-actin (anti-mouse ab8227, Abcam, Cambridge, MA) was used as a loading control. Western blots were repeated at least three times.

1. Hayama T, Yamaguchi T, Kato-Itoh M, Ishii Y, Mizuno N, Umino A, et al. Practical selection methods for rat and mouse round spermatids without DNA staining by flow cytometric cell sorting. Mol Reprod Dev. 2016;83(6):488-96. Epub 2016/04/01. doi: 10.1002/mrd.22644. PubMed PMID: 27031189.
